# Management of subtrochanteric femur fractures with internal fixation and recombinant human bone morphogenetic protein-7 in a patient with osteopetrosis: a case report

**DOI:** 10.1186/1752-1947-4-142

**Published:** 2010-05-19

**Authors:** Robert D Golden, Edward K Rodriguez

**Affiliations:** 1 Department of Orthopaedic Surgery, Beth Israel Deaconess Medical Center, 330 Brookline Avenue, Shapiro 2, Boston, MA, USA

## Abstract

**Introduction:**

Osteopetrosis is a group of conditions characterized by defects in the osteoclastic function of the bone resulting in defective bone resorption. Clinically, the condition is characterized by a dense, sclerotic, deformed bone which, despite an increased density observable by radiography, often results in an increased propensity to fracture and delayed union.

**Case Presentation:**

We report the case of a 27-year-old Asian man presenting with bilateral subtrochanteric femur fractures. He had a displaced right subtrochanteric femur fracture after a low-energy fall, which was treated surgically. The second fracture that our patient endured was diagnosed as a stress fracture ten weeks later when he complained of pain in the contralateral left thigh. By that time, the right-sided fracture exhibited no radiographic evidence of healing, and when the left-sided stress fracture was being treated surgically, bone grafting with recombinant human bone morphogenetic protein-7 was also performed on the right side.

**Conclusion:**

While there are no data supporting the use of bone morphogenic proteins in the management of delayed healing in patients with osteopetrosis, no other reliable osteoinductive grafting options are available to treat this condition. Both fractures in our patient healed, but based on the serial radiographic assessment it is uncertain to what degree the recombinant human bone morphogenetic protein-7 may have contributed to the successful outcome. It may have also contributed to the formation of heterotopic bone around the fracture site. Further investigation of the effectiveness and indications of bone morphogenic protein use for the management of delayed fracture healing in patients with osteopetrosis is warranted.

## Introduction

Osteopetrosis, originally described by Heinrich Albers-Schönberg in 1904 [1], is now known to be a group of conditions characterized by defects in osteoclastic function resulting in defective bone resorption [2]. Clinically, the condition is characterized by dense, sclerotic, deformed bones. Despite an increased density observable by radiography, it often results in an increased propensity for bones to fracture [3] and in problems with fracture healing [4]. We report the case of a patient with a history of osteopetrosis who first presented to our institution with an acute traumatic right subtrochanteric femur fracture and subsequently developed a stress fracture in a similar location in the contralateral extremity. To the best of our knowledge, this is the first reported case of the use of bone morphogenetic proteins (BMPs) in the treatment of a fracture in a patient with osteopetrosis.

## Case Report

Our patient was a 27-year-old Asian man with a history of osteopetrosis. He presented to the emergency department after slipping on ice, and reported twisting his right lower extremity before stabilizing himself with his left leg. He denied falling or striking his leg against anything. He complained of an immediate sharp pain in his right hip area, and was transported to the emergency department via ambulance.

Our patient had a known history of osteopetrosis and had previously suffered fractures in his left humerus, left tibia and right femur. All previous injuries had been managed non-operatively and healing had been prolonged. Our patient was not taking any medication and was not under continued management by any physician for his osteopetrosis.

On examination, our patient was afebrile with stable vital signs. His pelvis was stable and with no tenderness on palpation. His left hip was non-tender throughout a full range of motion. His right hip was tender to palpation and to any passive motion. The right lower extremity was neurologically intact to light touch sensation along all distributions. Motor function was intact in all lower extremity groups but our patient was unwilling to demonstrate the full range of movements of his right hip because of pain. All his extremities were well-perfused with palpable distal pulses.

**Figure 1:**
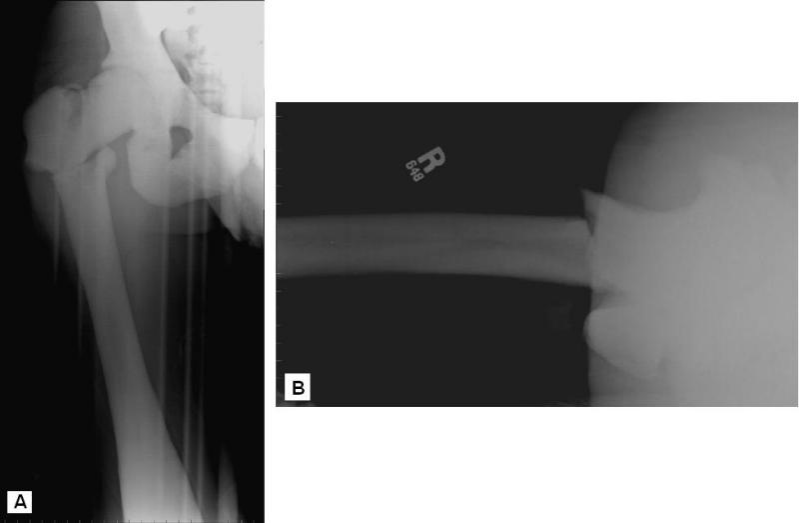
**Anteroposterior and lateral radiographs of our patient's right hip at the time of initial presentation to the emergency department**.

Radiographs revealed sclerotic bones consistent with osteopetrosis. X-rays of our patient's pelvis, right hip, right femur and chest were taken. There was a predominantly transverse fracture at the level of the lesser trochanter with a mild degree of posterior displacement of the distal fracture fragment (Figure 1). No left-sided subtrochanteric femur stress fracture was seen at this point.

Our patient was taken to the operating room the following morning for open reduction internal fixation with a right angle dynamic compression screw (DCS) implant (Synthes Inc, West Chester, PA, USA). The DCS implant was chosen as it could be used to apply effective axial compression along the transverse fracture and so to achieve absolute stability. A blade plate would have been an alternative implant but hammering the blade into place would have risked fracture propagation. Due to the extreme bone density of our patient and the lack of a femoral canal, the use of an intramedullary device was discounted. The procedure proved technically challenging due to the density and brittle nature of his bone. The use of numerous drill bits was required, with a prolonged drilling time. Constant irrigation and pauses were needed throughout the drilling process to prevent heat necrosis.

The drilling of the screw for the main lag screw of the DCS system proved to be particularly difficult and time-consuming. An eight-hole DCS plate with a 65 mm lag screw was used and this was secured to the femur with seven 4.5 mm cortical screws. The wound was closed over a drain and our patient was taken to the recovery room without incident. Intra-operatively, anatomic reduction with good compression was achieved at the fracture plane. Minimal dissection around the femur at the fracture site was carried out to minimize soft tissue stripping. The periosteum appeared to be normal in appearance.

**Figure 2:**
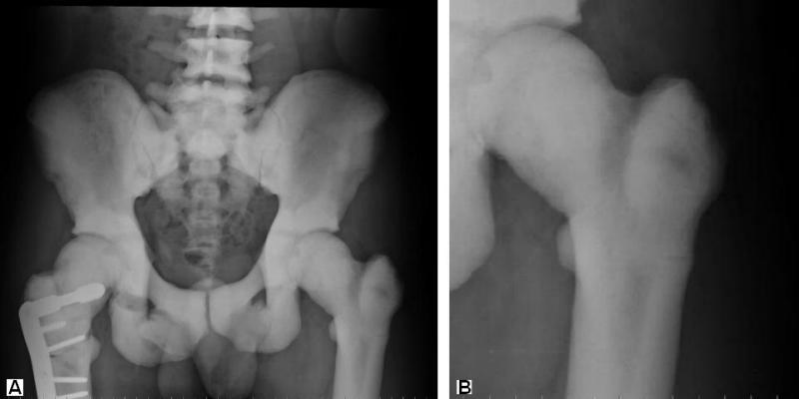
**(a) Anteroposterior radiograph of the pelvis obtained 10 weeks after initial fixation**. Note the lack of healing progression of the right femoral fracture and the stress fracture now visible in the left femur and better demonstrated in (b).

Our patient did well post-operatively and was discharged to a rehabilitation facility on his third day after surgery. He was initially allowed toe-touch weight bearing only on his operative side. At follow-up four weeks after his procedure, he was allowed to advance to 50% partial weight bearing. At that time, he continued to complain of some pain when walking, and discomfort while internally and externally rotating his hip. Radiographs demonstrated the hardware to be intact with satisfactory alignment of the fracture fragments but with no evidence of callus formation or other signs of progressive healing. Our patient returned for follow-up 10 weeks after his surgery and radiographs still failed to demonstrate any evidence whatsoever of a healing progression (Figure 2a). In addition, our patient reported contralateral upper thigh pain. A stress fracture on his contralateral femur was noted at the same level as the fracture managed operatively on the right side 10 weeks earlier (Figure 2b).

After discussion with our patient, it was recommended that he should return to the operating room to undergo internal fixation of the stress fracture in his left femur before it became a complete fracture like the one on the right side. In retrospect, there is radiographic evidence that the right-sided fracture was a stress fracture which had progressed to a displaced fracture after a relatively minor fall. Because our patient was going to have his left side fixed, and because of the poor progression of healing at this point, we also recommended that he underwent prophylactic bone grafting of the right side at the same time. We recommended the use of recombinant human bone morphogenetic protein-7 (rhBMP-7) to his right-sided fracture, citing his history of delayed healing of fractures, as evidenced by the slow healing from radiographs on his right side 10 weeks after the operation, as well as out of concern for early hardware failure in a relatively young and active patient.

The use of BMP graft was recommended in lieu of an iliac crest graft, given the expected difficulty of harvesting osteopetrotic crest as well as the questionable use of osteopetrotic bone as a useful grafting material. No data are available on the osteoinductive and osteoconductive properties of osteopetrotic bone. Furthermore, we feared that the process of harvesting iliac crest bone for grafting could put our patient at risk of a pelvic or acetabular fracture progression, given the brittle nature of his bone. He was informed that while rhBMP-7 has been approved for the treatment of nonunions in long bones, there were no data supporting its effectiveness in treating delayed long bone healing in patients with osteopetrosis. He agreed to the use of rhBMP-7 and informed consent was obtained. No plans were made to graft the left side, given that it was a newly diagnosed fracture. We did not seek approval from our Institutional Review Committee for the use of rhBMP-7 in a primary fracture which is currently not a Food and Drug Administration (FDA) approved procedure.

Our patient was brought to the operating room and underwent internal fixation of his stress fracture with a six-hole DCS construct utilizing a 60 mm lag screw. Once again, due to the extreme bone density of our patient, a prolonged drilling time with multiple drill bits was required. The right fracture site was grafted with a single dose of rhBMP-7 (OP-1^®^, Stryker Biotech, Hopkinton, MA, USA) through a smaller exposure along the original incision.

The post-operative course of our patient was unremarkable and he was discharged to a rehabilitation facility on post-operative day three. Follow-up radiographs obtained approximately six weeks after his second surgery demonstrated the hardware to be intact, but still no further healing of the initial fracture site on the right side was evident. Some early heterotopic bone formation was noted at the grafting site on the right side. At this point, our patient had weaned off of his postoperative pain control regimen consisting of 10 mg oxycodone/acetaminophen every four hours as need and was able to walk without a walker but felt safer with the support of a cane or a single crutch. He did, however, continue to complain of pain in his right hip during ambulation. Five and a half months after the operation, our patient continued to ambulate with a mildly antalgic gait. A computed tomography (CT) scan was carried out which showed some evidence of fracture healing, but the fracture lines were still visible at that time.

**Figure 3:**
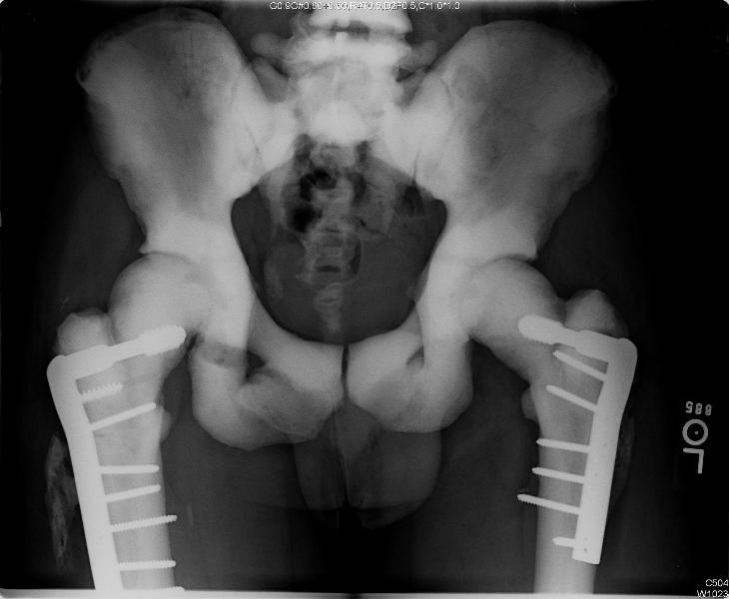
**Anteroposterior radiograph of the pelvis at the two-year follow-up appointment demonstrating attenuated but still visible fracture lines on the right side**.

Expectant management of our patient continued, and two years after his operations, radiographs continued to show visible, but less sharply defined fracture lines of his right hip (Figure 3). There was also evidence of mature heterotopic bone formation. There were no fracture lines visible on the left-sided stress fracture and there was no evidence of any loosening or hardware failure at either fracture site. Our patient walked with a non-antalgic gait and did not require any assistive devices. He had begun work as a dentist and stated that he could work all day on his feet without difficulty, but admitted to some fatigue with mild bilateral discomfort at the end of long days. His symptoms, however, were primarily focused at both groins and were more consistent with osteoarthritic pain. He neither ran nor participated in other athletic activities. He had 5/5 motor strength in all lower extremity muscle groups. He had not had any other orthopedic injuries since the time of his femur fractures.

Our patient moved out of our geographical area but kept in contact. About three years after his procedures, he contacted our office to request a referral to an orthopaedic surgeon closer to his new area of residence with experience of treating osteopetrosis. He was developing further osteoarthritic hip changes and needed advice regarding his options for arthroplasty versus resurfacing. Three years after his fractures, he had had no failure of fixation or other complications. He eventually underwent a total right hip arthroplasty at about 4 years from his initial fracture. We received a report that an initially planned resurfacing procedure was aborted due to the presence of femoral neck stress fractures. However, the subtrochanteric fracture was well healed. The left-sided fracture had no issues four years after its surgical management.

## Discussion

Osteopetrosis represents a group of conditions with defects in osteoclastic function resulting in defective bone resorption [2]. Armstrong, *et al*. conducted a survey using the members of the Pediatric Orthopedic Society of North America in an attempt to elucidate the optimal methods of treatment for fractures occurring in patients with osteopetrosis. Respondants cited various options for treatment but a consistent response from the participants regarded the difficulty in placing an internal fixation device secondary to the increased bone density.

Treatment without surgery was often reported to produce satisfactory results, although the rate of healing was frequently noted to be prolonged. Similar problems were seen with internal fixation, with case reports of delayed union of up to two years [4]. This is possibly due to the fact that the fracture callus in patients with osteopetrosis has been shown to be abnormal, with a continuation of unorganized woven bone and a lack of lamellar organization after one year, even in healed fractures [5]. A review of the osteopetrotic case reports by Birmingham and McHale [6] revealed a large variability in the healing rates of operatively-treated fractures, ranging from two months to two years, and with a few cases of nonunion. Actual case reports of nonunion in patients with osteopetrosis are rare in the literature [7,8].

Urist first described osteoinduction, later ascribed to BMPs, in 1965 [9]. There are now at least 18 different BMP molecules described, of which eight have been shown to have osteoinductive properties [10]. BMPs act on mesenchymal cells by inducing the recruitment of mesenchymal precursors from muscle and surrounding tissues into the fracture site, inducing osteoblast and chondrocyte differentiation, and inducing angiogenesis and eventual bone formation [11]. Currently, only two BMPs produced using recombinant gene technology have been commercialized: BMP-2 (Infuse^®^, Medtronic Sofamor, Minneapolis, MN, USA) and BMP-7 (OP-1^®^, Stryker Biotech, West Chester, PA, USA); and these are limited to approved applications in trauma and spinal fusion [12]. In particular, rhBMP-7 is approved for use in recalcitrant nonunions of long bones. Some studies have demonstrated that the use of BMPs is comparable with autografting in the treatment of nonunions without the morbidity associated with autograft harvesting and with a lower risk of infection than in those patients treated with autograft [10,12].

The osteoinductivity of BMPs also follows a dose-response ratio in which the local concentration of a BMP determines the clinical response. If the concentration is too low, inadequate bone formation will occur; if the dose is too high, heterotopic ossification might be expected, although this has not been shown to occur under physiological conditions, but it possibly does in osteopetrosis [13]. BMPs have also been shown to not only affect osteoblast activity, but also to stimulate osteoclast activity and osteoclastogenesis [14]. In fact, higher doses appear to often result in initial localized bone resoprtion [10]. However, it is unclear whether this effect will be seen in osteopetrotic bone with its underlying osteoclastic deficiency.

To the best of our knowledge, this is the first report of the use of BMP in the management of a femur fracture in a patient with osteopetrosis. Rafiq *et al*. reported the first case of the use of BMPs in osteopetrosis, referring to a humerus shaft fracture nonunion [8]. We report the results of a patient with bilateral femur fractures and osteopetrosis who was treated with internal fixation and rhBMP-7 grafting after his first fracture failed to show any signs of healing 10 weeks after an initial repair. During follow-up, our patient had some minor heterotopic bone formation on his right side and persistent radiographically apparent fracture lines suggesting some degree of incomplete healing at that site. However, there was no evidence of hardware failure suggesting a nonunion, and little discomfort and antalgia. Our patient also returned to full activity and remained complication-free on both sides for four years when a right hemiarthroplasty was performed for progressive hip osteoarthritis, at which time the hardware was removed and the fractures were confirmed to be healed. While we have no means of assessing to what extent the use of rhBMP-7 contributed to the healing process, and thus no firm evidence that it contributed at all, its use as an alternative to autologous bone graft in patients with osteopetrosis is an option for consideration. Further research to establish effectiveness and indications is warranted.

## Consent

Written informed consent was obtained from the patient for publication of this case report and any accompanying images. A copy of the written consent is available for review by the Editor-in-Chief of this journal.

## Competing interests

The authors declare that they have no competing interests.

## Authors' contributions

RDG examined the data, did the analysis and prepared the manuscript. EKR did the surgical intervention, conducted the post-operation follow-up, examined the data, did the analysis and prepared the manuscript. All authors read and approved the final manuscript.
